# Evolutionary significance of the microbial assemblages of large benthic Foraminifera

**DOI:** 10.1111/brv.12482

**Published:** 2018-11-18

**Authors:** Martina Prazeres, Willem Renema

**Affiliations:** ^1^ Marine Biodiversity Group Naturalis Biodiversity Center 2300 RA, Leiden, 9517 the Netherlands

**Keywords:** symbiosis, microbiome, coral reefs, climate change, ocean warming, Cenozoic

## Abstract

Large benthic Foraminifera (LBF) are major carbonate producers on coral reefs, and are hosts to a diverse symbiotic microbial community. During warm episodes in the geological past, these reef‐building organisms expanded their geographical ranges as subtropical and tropical belts moved into higher latitudes. During these range‐expansion periods, LBF were the most prolific carbonate producers on reefs, dominating shallow carbonate platforms over reef‐building corals. Even though the fossil and modern distributions of groups of species that harbour different types of symbionts are known, the nature, mechanisms, and factors that influence their occurrence remain elusive. Furthermore, the presence of a diverse and persistent bacterial community has only recently gained attention. We examined recent advances in molecular identification of prokaryotic (i.e. bacteria) and eukaryotic (i.e. microalgae) associates, and palaeoecology, and place the partnership with bacteria and algae in the context of climate change. In critically reviewing the available fossil and modern data on symbiosis, we reveal a crucial role of microalgae in the response of LBF to ocean warming, and their capacity to colonise a variety of habitats, across both latitudes and broad depth ranges. Symbiont identity is a key factor enabling LBF to expand their geographic ranges when the sea‐surface temperature increases. Our analyses showed that over the past 66 million years (My), diatom‐bearing species were dominant in reef environments. The modern record shows that these species display a stable, persistent eukaryotic assemblage across their geographic distribution range, and are less dependent on symbiotic photosynthesis for survival. By contrast, dinoflagellate and chlorophytic species, which show a provincial distribution, tend to have a more flexible eukaryotic community throughout their range. This group is more dependent on their symbionts, and flexibility in their symbiosis is likely to be the driving force behind their evolutionary history, as they form a monophyletic group originating from a rhodophyte‐bearing ancestor. The study of bacterial assemblages, while still in its infancy, is a promising field of study. Bacterial communities are likely to be shaped by the local environment, although a core bacterial microbiome is found in species with global distributions. Cryptic speciation is also an important factor that must be taken into consideration. As global warming intensifies, genetic divergence in hosts in addition to the range of flexibility/specificity within host–symbiont associations will be important elements in the continued evolutionary success of LBF species in a wide range of environments. Based on fossil and modern data, we conclude that the microbiome, which includes both algal and bacterial partners, is a key factor influencing the evolution of LBF. As a result, the microbiome assists LBF in colonising a wide range of habitats, and allowed them to become the most important calcifiers on shallow platforms worldwide during periods of ocean warming in the geologic past. Since LBF are crucial ecosystem engineers and prolific carbonate producers, the microbiome is a critical component that will play a central role in the responses of LBF to a changing ocean, and ultimately in shaping the future of coral reefs.

## I. INTRODUCTION

Across the globe, coral reefs are experiencing rapid declines due to deteriorating environmental conditions mainly driven by ocean warming (Pandolfi *et al.,* 2011; Hughes *et al.,* 2017). In these environments, symbiotic associations between organisms can provide the partners involved with the capacity to respond to environmental stresses as well as providing robustness under the challenges caused by climate change (Ainsworth & Gates, 2016). Associations with prokaryotic and eukaryotic microorganisms can facilitate the success of species across a variety of habitats, playing a fundamental role in the evolution and adaptive capacity of host organisms (Saffo, 1992; Cavanaugh, 1994), and have been associated with vulnerability when obligatory symbionts are expelled from their host (i.e. bleaching) (Hallock, 2000; Hughes *et al.,* 2017). Many heterotrophic organisms living in oligotrophic waters have evolved obligatory symbioses with photosynthetic microalgae, thus establishing biotrophic mixotrophy (Not *et al.,* 2016; Selosse, Charpin, & Not, 2017). This process is called photo‐symbiosis as it makes photosynthesis indirectly available to the host (Selosse *et al.,* 2017). However, mixotrophy comes at a cost, as it requires five times more energy and nutrient allocation to maintain the photosynthetic apparatus compared to maintaining a strictly heterotrophic feeding mode (Raven, 1997). Nonetheless, the development of mixotrophy allows organisms to occupy previously inaccessible niches, such as nutrient‐poor environments.

Photo‐symbiosis is critical to maintaining functioning coral reefs, not only in corals, the best‐known reef‐associated organisms, but also in the (often) overlooked unicellular eukaryotic large benthic Foraminifera (LBF). Symbiosis with eukaryotic taxa (i.e. microalgae) is essential for the health of reef ecosystems, and LBF are responsible for a significant proportion of the carbonate sediment across reef environments worldwide (Langer, 2008). From a global carbon perspective, LBF play a fundamental role in carbon sequestration and carbon cycling (Langer, Silk, & Lipps, 1997; Langer, 2008), in addition to sediment production and reef maintenance (Baccaert, 1986; Fujita & Fujimura, 2008; Dawson & Smithers, 2014; Dawson, Smithers, & Hua, 2014; Doo *et al.,* 2017). LBF species, especially those that produce high‐magnesium tests, serve an important role in maintaining the chemical equilibrium on coral reefs, serving to buffer against daily pH flux from reef metabolism through skeletal dissolution *post mortem* (Yamamoto *et al.,* 2012).

It is becoming increasingly apparent that other microorganisms such as bacteria and Archaea (henceforth referred to as prokaryotic associates), fungi, and viruses, play a significant and complex role in maintaining the host's health (Peixoto *et al.,* 2017). Prokaryotic microbial associations can benefit the host by enhancing nutrient cycling (S, C, and N), providing photosynthesis‐dependent nitrogen fixation, enhancing calcification, and in production of antimicrobials and pathogen removal (Knowlton & Rohwer, 2003; Lesser *et al.,* 2004). By contrast, the role of fungi and viruses remains elusive (Lecampionalsumard, Golubic, & Priess, 1995; Sweet & Bythell, 2017). Identifying specific microbes that provide critical functional contributions to a host organism requires an understanding not only of the endobiotic microbial population, but also of the persistence and stability in time and space of both the microbial functional niches and the microbes that utilise them (Ainsworth *et al.,* 2015; Hernandez‐Agreda *et al.,* 2016). These associations with microbial partners likely underpin the capacity of reef organisms to respond to climate change. Ocean warming will influence the biogeographic range of reef species, which could result in poleward expansion as subtropical and temperate marine ecosystems become ‘tropicalised’ (Verges *et al.,* 2014). The flexibility in these associations will determine the host's capacity to accommodate to local environmental change, as well as allowing adaptations to new environmental conditions following distribution range expansions.

The composition of both the prokaryotic microbiome and the eukaryotic symbionts in relation to environmental change has been explored largely in reef‐building corals (LaJeunesse, 2002; Ainsworth, Thurber, & Gates, 2010; Bourne, Morrow, & Webster, 2016). However, many other organisms, such as LBF, also benefit from the intricate interplay between prokaryotic endobionts and eukaryotic endosymbionts, mainly microalgae. Although only *ca*.10 families of benthic Foraminifera are currently known to have associations with algal symbionts, these families consist of many described species, which are abundant in shallow carbonate platforms worldwide. LBF are a polyphyletic group in which endosymbiosis with microalgae evolved independently in multiple benthic foraminiferal families (Hallock & Glenn, 1986; Hallock, 1999; Lee, 2011). The shell of LBF facilitates the housing of photosynthetic symbionts by morphological adaptations, including canaliculation, flosculisation, and the development of endo‐ and exoskeleton structures or secondary or lateral chamberlets (Renema, 2007). Evolutionary radiations indicate that the acquisition of, and change in, algal types were crucial steps in the evolution of large miliolid Foraminifera (Holzmann *et al.,* 2001). Symbiosis with algae has been suggested to be the driving force in the evolution of these groups of benthic Foraminifera (Lee, 2006, 2011; Lee *et al.,* 2010). LBF include members of two orders of foraminifera: Rotaliida and Miliolida (Hallock, 1999). The order Rotaliida, characterised by an optically radial, bilamellar perforate test (Pawlowski, Holzmann, & Tyszka, 2013), includes three modern families: Amphisteginidae, Calcarinidae, and Nummulitidae. The order Miliolida, with an imperforate wall, high‐magnesium calcite test and with randomly oriented crystals refracting light in all directions and resulting in a porcelaneous appearance of the test (Pawlowski *et al.,* 2013), includes the Alveolinidae, Peneroplidae, Soritidae, and Archaiasidae (Loeblich & Tappan, 1984). In general, Rotaliida species are known predominantly to host diatoms, whereas Milioliida also play host to other algal groups, such as chlorophytes, rhodophytes, and dinoflagellates (Lee, 2006). Additionally, modern species of both groups have associations with a diverse prokaryotic community, including heterotrophic bacteria, photosynthetic cyanobacteria, and algal plastids, suggesting that Foraminifera are particularly favourable partners for the establishment of symbioses (Lee, 2006; Bourne *et al.,* 2013).

At least 47 modern species across 15 genera have been identified as possessing algal symbionts (Lee, 2006; Förderer, Rödder, & Langer, 2018). Whereas eukaryotic symbiosis has received considerable attention, prokaryotic symbiosis and the role of bacteria in LBF remains largely unexplored (e.g. Webster *et al.,* 2016; Prazeres *et al.,* 2017a; Prazeres, 2018). Not only is the diversity of bacterial communities poorly known, but so is the relationship and role that these bacteria may have in LBF ecology, adaptive potential, and evolution. In this review, we aim to determine how the eukaryotic and prokaryotic microbiome influences the capacity of LBF to occupy new habitats, expand their distribution range, and adapt or acclimatise to shifts in environmental conditions. For the purposes of this review, eukaryotic symbionts and prokaryotic partners are considered algal and bacterial species, respectively. Firstly, we explore the known algal partners and how they influence modern LBF species' biology and ecology. We will also discuss the geographical distribution of fossil LBF species within their environmental context, and link it to their microbiome, particularly to algal symbionts. Finally, we argue that the microbiome (i.e. algal and bacterial species) is likely to be crucial in the resilience, acclimation, and adaptation of LBF in the face of climate change.

We discuss how the microbiome could benefit and drive LBF evolution across species distribution ranges: (*i*) by persistent eukaryotic and prokaryotic microbial associations across the distribution of LBF species, which have been reported to be highly species‐specific and to determine ecological niches in LBF; (*ii*) the presence of a variable microbiome responsive to environmental gradients; (*iii*) the presence of a stable, persistent algal symbiont community coupled with flexible bacterial associations; and (*iv*) adaptable algal symbiosis with a persistent bacterial community, which could assist species in crossing biogeographical barriers and adapting to changing environmental conditions. The composition of the microbiome benefits the host in different ways, and different species are likely to utilise different strategies to maintain the holobiont system. Therefore, it is crucial to understand and disentangle how the host and symbiont compartments are likely to interact with, and respond to environmental change.

## II. EVOLUTION OF ALGAL SYMBIOSIS IN LARGE BENTHIC FORAMINIFERA

Symbioses are often cited as a pathway for the abrupt appearance of evolutionary novelty, and as facilitating the occupation of habitats and niches previously unavailable to asymbiotic counterparts (Norris, 1996; Melo Clavijo *et al.,* 2018). Throughout Foraminifera evolution, the recurrent rise of algal symbiosis coincides with periods of global warming, relative drought, high sea levels, and the expansion of tropical and subtropical belts into higher latitudes (Lee, 1995; Boudagher‐Fadel, 2008). In benthic Foraminifera, the presence of algal symbiosis in fossil forms is deduced mainly through the study of morphological adaptations, such as extrapolation of test size and shape, chamber arrangement, and ultrastructural modifications to regulate light intensity to the photosymbionts (Renema, 2018). Measurement of growth rates and patterns of stable isotopes have also been used to recognise algal symbiosis in the fossil record (Erez, 1978; Purton & Brasier, 1999; Briguglio, Metscher, & Hohenegger, 2011; Briguglio, Hohenegger, & Less, 2013). Taxonomic radiation and acquisition of photosymbiosis allowed shifts in ecological strategy, enabling benthic Foraminifera to expand into a variety of habitats and to become abundant in oligotrophic environments (Hallock, 1985).

**Figure 1:**
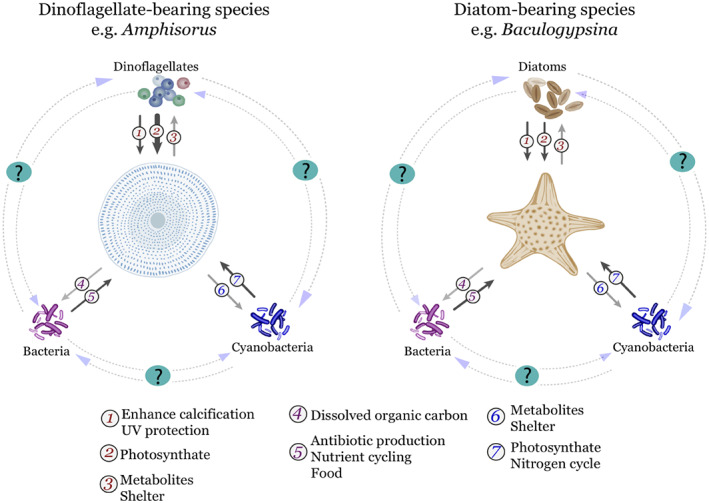
Possible roles and relationships between large benthic Foraminifera and their algal symbionts and bacterial groups. Dinoflagellate‐bearing species are more dependent on their symbiont than diatom‐bearing and other algal‐bearing species for acquiring energy. Therefore, it is likely that species that rely less on algal symbiosis for growth and calcification utilise bacteria as a food source and require additional translocation of organic compounds from cyanobacteria. Bold arrows correspond to a high dependence on the exchange represented. Light grey arrows represent compounds being exchanged from the host to the microbial associate, whereas dark grey arrows represent the exchange from the microbial associate to the host.

The association with algal symbionts resulted in a mixotrophic lifestyle, allowing the utilisation of both inorganic and organic sources of nutrients for photosynthesis and organic carbon accumulation necessary for metabolism and reproduction (Hallock, 1981). Morphological modifications in benthic Foraminifera resulted in adaptations of internal structures, allowing the test to evolve to accommodate their algal symbionts (Lee & Hallock, 1987). Algal symbiosis is particularly advantageous in environments where light is readily available, dissolved inorganic dissolved nutrients are scarce, and significant amounts of energy must be expended to capture organic matter (Hallock, 1981; Lee, 1995). Therefore, the direct benefits of photosymbionts are twofold: (*i*) the acquisition of energy through photosynthesis, which is particularly favourable in oligotrophic environments (Hallock, 1981); and (*ii*) enhanced calcification rates, because the energy acquired through photosynthesis is orders of magnitude higher than in heterotrophy alone (Hallock, 1981; ter Kuile, 1991). Additionally, hosts are protected against ultraviolet (UV) irradiation by housing algae as symbionts, which can minimise the effects of hazardous irradiation through the production of photo‐protective amino acids by the symbionts (Hohenegger, 2009; Fig. 1). These morphological and physiological modifications were fundamental to the successful acquisition of a wide range of microbial associates by LBF.

Members of various modern LBF families are hosts to a variety of algal symbionts (Stanley & Lipps, 2011). Even though most LBF hosts are mixotrophic, they usually cannot survive for long periods without their endosymbiotic algae (Lee, 2006). Cyanobacteria and bacteria are also suggested to be important in LBF biology and ecology (Lee & Anderson, 1991; Bernhard *et al.,* 2012). Bacteria can function as antibiotic producers, perform nutrient cycling, and be ingested as food when other types of organic matter are not available, while cyanobacteria can provide additional photosynthetic products to the host when light is available (Fig. 1). This symbiont diversity is in sharp contrast to many reef‐building corals, which are only capable of hosting dinoflagellates (Muscatine & Porter, 1977; Stanley, 2006; Stanley & Lipps, 2011). LBF are the only known taxa that both feed on particulate food and are able to sustain symbiosis with diatoms, which are one of the most common microalgae (Lee & Anderson, 1991). The benefits from symbiosis and the capacity to accommodate different types of endosymbionts have facilitated adaptation of LBF to a variety of environments, with varied regimes of light, temperature, salinity, and nutrients, which will be discussed in detail below.

## III. DIVERSITY AND ECOLOGICAL IMPORTANCE OF ALGAL SYMBIONTS

**Figure 2:**
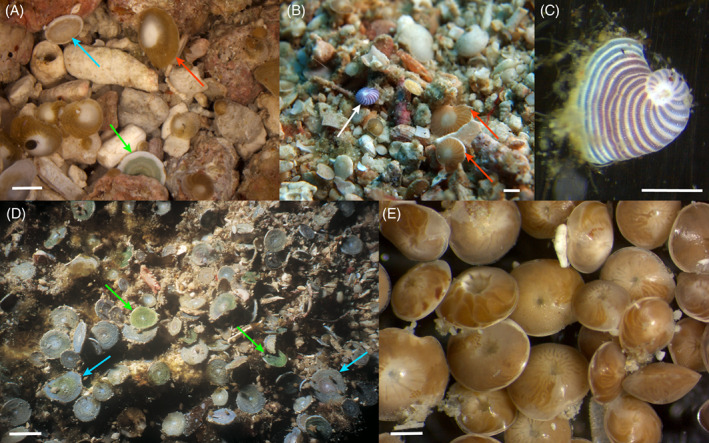
Different species of large benthic Foraminifera host diverse algal symbiont communities. (A) Diatom‐bearing, dinoflagellate‐bearing and chlorophyte‐bearing species in sediment samples from Heron Island, southern Great Barrier Reef, Australia, represented by red, green, and blue arrows, respectively (scale bar = 0.5 cm). (B) Rhodophyte‐bearing *Dendritina* sp. (white arrow), and diatom‐bearing specimens of *Operculina* and *Nummulites* (red arrows) found in reefs off Spermonde Archipelago, Indonesia (scale bar = 1 cm). (C) Rhodophyte‐bearing *Peneroplis planatus* collected at Lizard Island, northern Great Barrier Reef, Australia (scale bar = 250 μm). (D) Specimens of *Amphisorus* sp. from Mindanao, Philippines, hosting symbiotic chlorophytes (green arrows) and dinoflagellates (blue arrows) (scale bar = 1 mm). (E) Diatom‐bearing *Amphistegina lobifera* and *Amphistegina lessonii* from the northern Great Barrier Reef, Australia (scale bar = 0.5 mm).

**Figure 3:**
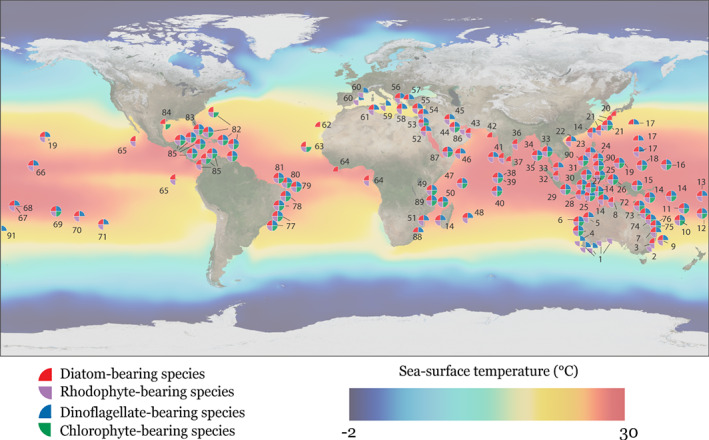
Modern global distribution of large benthic Foraminifera with algal symbionts, and average sea‐surface temperature. 1, Li *et al*. (1999); 2, Smith *et al*. (2001); 3, Albani (1978); 4, Betjeman (1969); 5, Orpin, Haig, & Woolfe (1999); 6, Parker (2009); 7, Narayan & Pandolfi (2010); 8, Michie (1987); 9, Heron‐Allen & Earland (1924); 10, Debenay (2012); 11, Kosciuch *et al*. (2018); 12, Todd (1965); 13, Collen & Garton (2004); 14, W. Renema, personal observation; 15, Langer & Lipps (2003); 16, Fujita *et al*. (2009); 17, Lessard (1980); 18, Makled & Langer (2011); 19, Hallock (1984); 20, Oki (1989); 21, Sugihara, Masunaga, & Fujita (2006); 22, Zheng & Zheng (1978); 23, Hallock & Glenn (1986); 24, Renema (2002); 25, Hoefker (1927); 26, Natsir & Subkhan (2012); 27, Renema & Troelstra (2001); 28, Renema (2003); 29, Renema (2008a); 30, Burollet *et al*. (1986); 31, Renema (2006a); 32, Natsir, Subkhan, & Wardhani (2012); 33, Jumnongthai (1980); 34, Jayaraju, Reddy, & Reddi (2011); 35, Muruganantham, Ragavan, & Mohan (2017); 36, Vedantam & Rao (1970); 37, Jayaraju & Reddi (1996); 38, Pisapia *et al*. (2017); 39, Parker & Gischler (2011); 40, Murray (1994); 41, Bhalla *et al*. (2007); 42, Rao (1971); 43, Pilarczyk *et al*. (2011); 44, Clarke & Keij (1973); 45, Parker & Gischler (2015); 46, Al‐Wosabi, Mohammed, & Al‐Kadasi (2011); 47, Hottinger (1980); 48, Karisiddaiah, Veerayya, & Guptha (1988); 49, Thissen & Langer (2017); 50, Zinke *et al*. (2005); 51, Langer *et al*. (2013a); 52, Haunold, Baal, & Piller (1997); 53, Hottinger, Halicz, & Reiss (1993); 54, Hyams, Almogi‐Labin, & Benjamini (2002); 55, Mouanga & Langer (2014); 56, Koukousioura, Dimiza, & Triantaphyllou (2010); 57, Hollaus & Hottinger (1997); 58, Triantaphyllou, Koukousioura, & Dimiza (2009); 59, Caruso & Cosentino (2014); 60, Langer *et al*. (2012); 61, El Kateb *et al*. (2018); 62, Pascual & Martín‐Rubio (2004); 63, Lévy *et al*. (1997); 64, Fajemila & Langer (2017); 65, McCulloch (1981); 66, Ebrahim (2000); 67, Cushman (1924); 68, Fajemila, Langer, & Lipps (2015); 69, Fujita & Omori (2015); 70, Bicchi, Debenay, & Pages (2002); 71, Whittaker & Hodgkinson (1995); 72, Yamano, Miyajima, & Koike (2000); 73, Baccaert (1987); 74, Renema, Beaman, & Webster (2013); 75, Jell, Maxwell, & McKellar (1965); 76, Mamo (2016); 77, Araújo & Machado (2008); 78, Barbosa *et al*. (2012); 79, Lévy *et al*. (1995); 80, Batista, Vilela, & Koutsoukos (2007); 81, Machado & Souza (2017); 82, Javaux & Scott (2003); 83, Cockey, Hallock, & Lidz (1996); 84, Culver & Buzas (1981); 85, Culver & Buzas (1982); 86, Al‐Wosabi, Mohammed, & Basardah (2017); 87, Murray (1974); 88, Förderer, Rödder, & Langer (2018); 89, Heron‐Allen & Earland (1915); 90, Cushman (1921); 91, Hayward *et al*. (1999).

LBF host a diverse array of algal symbionts (Fig. 2; Table 1). Most higher taxonomic groups predominantly host a single type of symbiont. Within the algal groups known to form symbiosis with LBF, most modern and fossil species host diatoms. Only the clade Soritacea hosts a variety of different algal types. The Soritacea form a monophyletic group and have evolved from an asymbiotic lifestyle into symbiosis with rhodophytes and chlorophytes, and later dinoflagellates (Leutenegger, 1984; Holzmann *et al.,* 2001; Fay, 2010). The basal clade, Peneroplidae, host unicellular rhodophytes (Lee, 2006), Archaiasidae and *Parasorites*, which is the most basal genus in the Soritidae, host chlorophytes (Pawlowski *et al.,* 2001a), and all other Soritidae host dinoflagellates (Pawlowski *et al.,* 2001b). The depth distribution of LBF taxa is partly determined by the light intensity and wavelengths required by their symbionts (Renema, 2018). Light intensity and spectrum are important factors limiting the distribution of the host species at the lower end of their depth range, while the upper end of the range is determined by additional factors, such as wave energy (Hottinger, 1983; Hohenegger *et al.,* 1999; Renema, 2018). There is a progressive increase in the maximum depth of occurrence from chlorophyte‐bearing species, through species hosting rhodophytes or dinoflagellates, to species harbouring diatoms, which are distributed over the largest depth range (Leutenegger, 1984). The geographic range limits are often determined by temperature gradients, limiting their occurrence to the (sub)tropical to warm temperate regions (Langer & Hottinger, 2000; Renema, 2018). The occurrence of LBF at their highest latitudes is associated with warm boundary currents (e.g. Kuroshio in the northwest Pacific, the Leeuwin current in the east Indian Ocean, and the Gulf Stream in the northeast Atlantic; Fig. 3).

**Table 1:** List of extant LBF taxa cited in the text, and their respective major algal symbionts

Order	Family	Genus	Species	Major algal symbiont group
Rotaliida	Amphisteginidae	*Amphistegina*	*A. gibbosa* *A. lessonii* *A. lobifera* *A. radiata*	Diatoms
	Nummulitidae	*Cycloclypeus*	*C. carpenter*	
		*Heterostegina*	*H. depressa*	
		*Nummulites*	*Nummulites* sp.	
		*Operculina*	*Operculina* sp.	
	Calcarinidae	*Baculogypsina*	*B. sphaerulata*	
		*Calcarina*	*C. gaudichaudii* *C. hispida*	
		*Pararotalia*	*P. calciformata*	
Miliolida	Archaiasidae	*Archaias*	*A. angulatus*	Chlorophytes
	Soritidae	*Parasorites*	*Parasorites* sp.	
		*Amphisorus*	*A. hemprichii*	Dinoflagellates
		*Marginopora*	*M. vertebralis*	
	Peneroplidae	*Dendritina*	*Dendritina* sp.	Rhodophytes
		*Peneroplis*	*P. planatus* *P. pertusus*	

**Figure 4:**
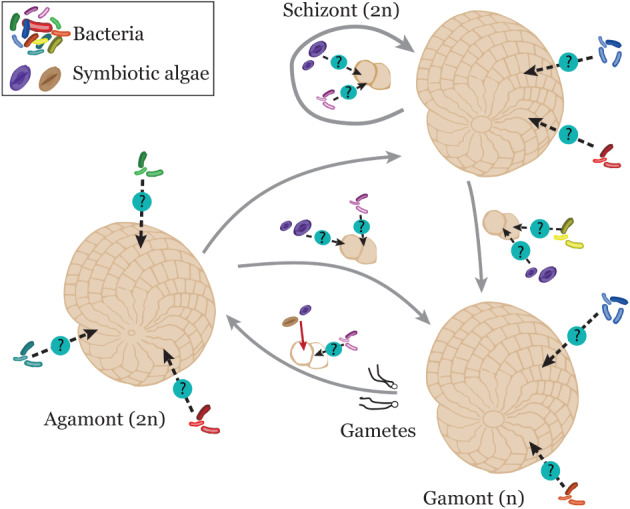
The typical life cycle of large benthic Foraminifera showing potential routes for acquisition of algal symbionts and bacteria. During sexual reproduction gametes do not carry the algal symbionts, and symbionts are acquired horizontally. By contrast, algal symbionts are vertically acquired during asexual fission. It remains unclear if adults acquire algal symbionts and bacteria from the environment, and how bacteria are transferred from parents to offspring. Dashed black arrows denote uncertain routes of acquisition and the solid red arrow denotes a known transfer route.

Algal symbionts can be acquired from the environment or through reproduction, and LBF utilise different strategies to pass on the algal symbiont to their offspring. LBF have a paratrimorphic life cycle, with both asexual and sexual reproductive phases (Lee *et al.,* 1997; Dettmering *et al.,* 1998; Harney, Hallock, & Talge, 1998). A paratrimorphic life cycle distinguishes three generations (Fig. 4): (*i*) agamonts, which are diploid, multinucleate, and microspheric (i.e. with a small initial chamber of the test); (*ii*) schizonts that are diploid, multinucleate, and megalospheric (i.e. with a large initial chamber of the test); and (*iii*) gamonts, which are haploid, mononucleate and megalospheric (Fig. 4) (Dettmering *et al.,* 1998). The sequence of the three generations in a paratrimorphic life cycle is not obligatory, thus offering foraminifera potential benefits in terms of flexibility (Harney *et al.,* 1998). LBF can transmit their symbionts vertically through rounds of asexual reproduction (megalospheric forms), and horizontally through rounds of sexual reproduction (microspheric and megalospheric forms) (Harney *et al.,* 1998). By optimising these different symbiont acquisition strategies, LBF can potentially increase their capacity to adapt to new environmental conditions. However, the mechanisms that affect the horizontal transfer of eukaryotic symbionts following sexual reproduction remain unclear. Free‐living representatives of eukaryotic foraminiferal algal endosymbionts are rare (Lee, 2006). Therefore, algal symbionts are possibly acquired immediately after reproduction by taking in symbionts expelled from the parent. Alternatively, there may be insufficient data available on the diversity and abundance of free‐living species that could be potential LBF symbionts. We now review the available data on the four main types of algal endosymbiont taxa found in modern LBF, and their role in LBF biology and ecology.

### (1) Class Bacillariophycea (Diatoms)

Four independently evolved families of LBF (Alveolinidae, Calcarinidae, Amphisteginidae, and Nummulitidae) host endosymbiotic diatoms (Hallock, 1999). It is worth noting that an additional non‐LBF family, the Elphidiidae, includes species that can sequester photosynthetically active plastids from diatoms (Correia & Lee, 2000, 2002a, 2002b; Jauffrais *et al.,* 2018). All endosymbiotic diatoms within LBF share a 104 kDa glycoprotein on their surface, which is not found on the surface of free‐living diatoms (Lee & Reyes, 2006). On substrates where species of LBF are found, free‐living endosymbiotic diatoms represent less than 0.5% of the microflora (Lee *et al.,* 1989), further indicating that both host and algae are involved in an obligatory, mutualistic relationship. As a result, loss of symbionts (i.e. bleaching) can lead to reduced growth, oxidative stress, fecundity impairment, and increased mortality of host populations (Prazeres, Martins, & Bianchini, 2012; Prazeres, Roberts, & Pandolfi, 2017b).

Early morphological studies of diatom symbionts were based on culturing isolated symbionts from LBF cell material (Lee *et al.,* 1993; Lee & Correia, 2005). This technique demonstrated that diatom symbionts are diverse and include around 20 identified species of pennate diatoms (Chai & Lee, 2000; Lee *et al.,* 1993, 1989, 1995, 2000). Species of the genera *Nitzschia*, *Nanofrustulum*, *Amphora*, and *Navicula* were the most commonly observed diatom symbionts in *Amphistegina* spp., whereas *Achnanthes* is a diatom genus commonly associated with *Heterostegina* (Lee *et al.,* 1989). Morphological studies showed that while LBF commonly host a single dominant symbiont species at any given time, several other diatom species can be present at low abundances (Lee *et al.,* 1989), and some flexibility in these host–symbiont associations is usually observed. However, molecular studies demonstrate that this diversity is likely to have been underestimated by morphological studies (Holzmann, Berney, & Hohenegger, 2006; Prazeres *et al.,* 2017a). Molecular studies of diatom symbionts in LBF also suggest a strong, species‐specific host–symbiont relationship (Holzmann *et al.,* 2006; Schmidt *et al.,* 2015, 2018; Barnes, 2016; Prazeres *et al.,* 2017a). Diatom‐bearing species also have associations with other groups of algae such as the green microalga *Chlorella* (Lee, 2006), and possibly with rhodophytes, usually at very low relative densities (Prazeres *et al.,* 2017a). However, given their mixotrophic nature, LBF could be utilising these other algal groups as food sources. Further studies investigating the intracellular location of these minor components are needed to distinguish between symbiotic partners and those contained in food vacuoles.

**Figure 5:**
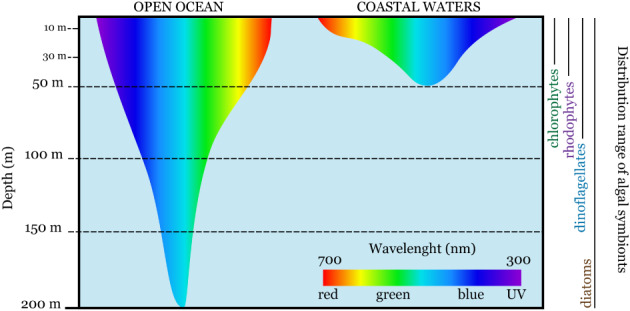
Light penetration in oceanic and coastal waters, and the known vertical distribution of algal symbionts in large benthic Foraminifera.

Diatom‐bearing species show the broadest depth and latitudinal range distribution among the LBF. They are common and abundant in tropical and subtropical areas (Langer & Hottinger, 2000; Langer *et al.,* 2013b; Weinmann *et al.,* 2013b). Diatom‐bearing species require mainly blue–green spectrum light to photosynthesise (Leutenegger, 1984) and, as a result, can colonise deeper areas of open ocean (>100 m) where only blue light is able to penetrate (Fig. 5). For example, members of the Amphisteginidae and Nummulitidae families can live at over 130 m water depth or <1% surface photosynthetically active radiation (PAR) (Hallock & Peebles, 1993; Hohenegger, 2000; Renema, 2006b *;* Boudagher‐Fadel, 2008). However, other requirements besides the light spectrum, such as light intensity, substrate and wave energy, also affect their distribution (Renema, 2018). A field colonisation experiment showed that diatom‐bearing species, mainly amphisteginids and nummulitids, avoid high light levels, and are often found in shaded microhabitats (Fujita, 2004). By contrast, the shallow occurrence of calcarinids, for example, is presumably not only linked with the symbionts' requirements for high light levels, but also with their capacity to withstand elevated hydrodynamic stress, as these species possess the ability to attach firmly to substrates (Leutenegger, 1984; Fujita, 2004, 2008). Moreover, some species of calcarinids can live in mesotrophic conditions and are abundant on inshore, turbid reefs of the West Pacific Ocean (Renema & Troelstra, 2001; Renema, 2010). Tolerance limits vary among species, and the mechanisms through which some species such as *Calcarina hispida* are able to replace more‐sensitive taxa such as *Amphistegina lobifera* (Renema, 2010) remain unclear, as both species harbour diatoms as symbionts, have similar ecological requirements, and occupy the same reef habitat. It is possible that the underlying difference between species' tolerance to mesotrophy and eutrophy hinges on their prokaryotic microbiome, and the extent to which they depend on heterotrophy for energy intake.

In summary, diatom‐bearing species can be broadly divided into two groups: high‐ and low‐light adapted. High‐light‐adapted species inhabit mainly well‐lit reef flat areas, whereas the low‐light adapted group shows cryptic behaviour and is commonly found along the reef slope. The ability of diatom‐bearing LBF to inhabit this broad range of environments can be explained by (*i*) their ability to host multiple species and strains of diatom symbionts (Lee *et al.,* 1995), and potentially benefit from associations with other algal groups; and (*ii*) their remarkable morphological plasticity (Hallock, Forward, & Hansen, 1986; Prazeres & Pandolfi, 2016; Prazeres, Uthicke, & Pandolfi, 2016a), which allows LBF to regulate light intensity to their symbionts, avoiding photo‐inhibition and damage to the photosystem (Hottinger, 1983).

### (2) Class Dinophyceae (Dinoflagellates)

The Soritidae are the only LBF family that house dinoflagellate symbionts (Fay, 2010), with the exception of the basal genus *Parasorites*, which hosts chlorophytes (Holzmann *et al.,* 2001; Pawlowski *et al.,* 2001a). *Symbiodinium* is the most common genus of dinoflagellates in soritids, but other less‐abundant dinoflagellate species have also been isolated (Lee & Anderson, 1991). It is worth noting that planktonic Foraminifera species are hosts to this same genus of dinoflagellates (Lee & Anderson, 1991). Dinoflagellates of the genus *Symbiodinium* are crucial components of coral reef ecosystems in their roles as endosymbionts of reef‐building corals (Muscatine & Porter, 1977) and other marine invertebrates, such as molluscs, sponges, and other cnidarians (LaJeunesse, 2002; Stat, Carter, & Hoegh‐Guldberg, 2006). Molecular phylogenetic studies have revealed an extraordinary diversity of *Symbiodinium* lineages, most of which are specifically associated with this group of foraminifera (Pochon *et al.,* 2001; Pochon & Pawlowski, 2006). The genus *Symbiodinium* encompasses nine lineages, delineated phylogenetically using nuclear and chloroplast ribosomal DNA and referred to as clades A to I (Pochon, LaJeunesse, & Pawlowski, 2004; Pochon & Gates, 2010).

Geographic variation in the distribution of *Symbiodinium* clades in soritids, such as the absence of *Symbiodinium* clade C in the Caribbean population of *Sorites* and its presence in the population of the same phylotype of *Sorites* on the Pacific side of the Isthmus of Panama (Pochon *et al.,* 2004), suggests that these symbiotic associations have evolved in response to the different environments of each region over the last 3–4 million years (My) (Garcia‐Cuetos, Pochon, & Pawlowski, 2005). It has been suggested that soritids have strong host–symbiont specificity (Pochon *et al.,* 2001), resulting from a combined effect of a selective recognition mechanism, vertical transmission of symbionts, and biogeographical isolation (Garcia‐Cuetos *et al.,* 2005). Nonetheless, mixed infections have been observed (Pochon *et al.,* 2007; Momigliano & Uthicke, 2013), and hosts can compartmentalise symbionts (Fay, 2010). In some soritids, such as *Marginopora vertebralis*, *Symbiodinium* diversity can be high (up to four different clades) in marginal habitats characterised by high seasonal fluctuations in environmental parameters (Momigliano & Uthicke, 2013). While symbiont polymorphism seems to be a common phenomenon, a higher diversity of mixed genotypes is observed more frequently in juvenile specimens, which may be more able to switch or shuffle heterogeneous symbiont communities than adults (Pochon *et al.,* 2007). During ontogeny, symbiont diversity decreases, as their *Symbiodinium* community moves towards species suited to the prevailing environmental conditions (Pochon *et al.,* 2007).

Dinoflagellate‐bearing species can colonise a wide range of reef habitats, and occur at depths of up to 80 m when conditions allow optimal light penetration, but are most abundant and commonly found on reef flats and upper reef slopes above 5 m, where light levels are highest (Hohenegger *et al.,* 1999; Renema & Troelstra, 2001; Doo *et al.,* 2017). These species have a narrower geographical distribution than diatom‐bearing LBF and are more diverse in the Indo‐Pacific Ocean. The genera *Sorites* and *Amphisorus* are circumtropical, and are the only genera to occur in the West Atlantic Ocean, whereas *Marginopora* is limited to the West Pacific Ocean (Fay, 2010). Light preference varies among species. For example, *Marginopora vertebralis* is adapted to high light, and is abundant in shallow reef areas (Pochon *et al.,* 2007). However, species such as *Sorites orbiculus* and *Amphisorus hemprichii* are common in low‐light environments, and can be found in deeper regions and shaded micro‐habitats on the reef flat (Hohenegger, 2000).

It appears that dinoflagellate‐bearing hosts can maintain mixed infections, and preferentially select symbionts from the available species pool in order to respond to changes in environmental conditions. As opposed to diatom‐bearing species, dinoflagellate‐bearers have the advantage of selecting from a higher diversity of symbionts given that other reef organisms are also hosts of *Symbiodinium*, such as sponges, reef‐building corals, and molluscs (Coffroth & Santos, 2005). Even though this group of LBF are relatively dependent on their symbionts, other reef organisms can act as reservoirs of symbiont diversity.

### (3) Division Chlorophyta

The chlorophyte‐bearing foraminifera comprise at least 13 species classified into five genera (Lee, 2006). They all belong to the subfamily Archaiasinae, with the exception of the soritid genus *Parasorites*. Most of these species are endemic to the Western Atlantic. A few species, including *Parasorites orbitolitoides* and *Laevipeneroplis malayensis*, have been reported from the Indo‐Pacific (Renema, 2007; Muruganantham *et al.,* 2017). Additionally, *Parasorites* sp. has been observed to host symbionts other than chlorophytes (Renema, 2003).

Chlorophyte‐bearing species have a narrower depth distribution than those bearing diatoms and dinoflagellates. Chlorophyte endosymbionts require primarily red light to photosynthesise (Fig. 5) and lack accessory pigments to allow them to absorb light in the blue region of the spectrum (Lee & Hallock, 1987). Chlorophyte‐bearing LBF represent a highly diverse group and can be found across a wide range of shallow habitats and environmental conditions. These species are particularly diverse in the Western Atlantic and Caribbean (Hallock, 1999), and are abundant in shallow environments (<30 m). For example, in the Florida Keys, *Androsina lucasi* can be found in exceptional abundance in open, dwarf‐mangrove flats at less than 0.2 m depth. *Archaias angulatus* live at depths less than 2 m, where temperatures range from 14°C in winter to 33°C in summer. *Cyclorbiculina compressa*, *Parasorites orbitolitoides*, *Laevipeneroplis proteus*, and *L. bradyi* inhabit a broader depth range of 5–30 m (Hallock & Peebles, 1993).

Molecular and morphological data show that the chlorophyte symbionts of LBF belong to the genus *Chlamydomonas* (Pawlowski *et al.,* 2001a *;* Lee, 2006). Like diatom‐ and dinoflagellate‐bearing species, chlorophyte‐bearing species seem to have exceptionally flexible relationships with their symbionts (Lee *et al.,* 1997). All foraminiferal symbionts form a monophyletic group closely related to *Chlamydomonas noctigama* (Pawlowski *et al.,* 2001a). The group is composed of seven types, including *C. hedleyi* and *C. provasoli*. Each of these types can be considered a separate species, based on comparisons of genetic differences between other established *Chlamydomonus* species (Pawlowski *et al.,* 2001a). Several LBF species share the same symbiont type, but only *Archaias angulatus* has been observed to host more than one chlorophyte species (Pawlowski *et al.,* 2001a). Symbionts of all Caribbean and Indo‐Pacific genera studied to date are closely related, suggesting a single origin of symbiosis between chlorophytes and Soritacea (Holzmann *et al.,* 2001; Pawlowski *et al.,* 2001a). In the Red Sea, chlorophytes have also been isolated as minor endosymbionts or intracytoplasmic associates from *Amphistegina* spp. and *Amphisorus hemprichii*, which host mostly diatoms and dinoflagellates, respectively (Lee & Anderson, 1991).

Chlorophyte‐bearing species are less dependent on their symbionts for energy acquisition, and can be found in relatively productive coastal waters (Walker *et al.,* 2011). Some species, such as *Archaias angulatus*, are known to tolerate relatively high levels of nutrients, and feeding provides 10× more carbon than carbon fixation through symbiotic photosynthesis (Lee & Bock, 1976). Nonetheless, symbiosis enhances calcification in chlorophyte‐bearing species (Duguay & Taylor, 1978). Hosts are unable to grow in very low light environments, but will quickly reach maximum photosynthesis rates under high light intensity (Walker *et al.,* 2011).

### (4) Division Rhodophyta

Members of the family Peneroplidae, including the genera *Peneroplis*, *Dendritina*, *Spirolina*, and *Monalysidium* (Loeblich & Tappan, 1984) are known to host red algae. As opposed to other symbiont algal groups, diversity of species of rhodophytes that form symbioses with LBF are relatively poorly known. To date, morphological studies identified only one symbiotic species, *Porphyridium purpurum*, which has been isolated from both *Peneroplis planatus* and *P. pertusus* (Lee, 1990). The isolated strains are conspecific (Lee, 1990) although molecular studies are yet to be carried out to confirm the identity of this species and perhaps reveal a higher diversity of endosymbiotic rhodophytes. Similarly, to chlorophyte‐bearing species, rhodophyte‐bearers generally have a narrow depth distribution. They are most common and abundant between 0 and 20 m and require primarily yellow/orange light for photosynthesis (Fig. 5) (Hohenegger *et al.,* 1999; Boudagher‐Fadel, 2008), but can occasionally be found in deeper areas, as deep as 40 m, of the reef system (Renema, 2018).

The relationship between rhodophytes and Foraminifera is unique. Unlike other eukaryotic symbionts in LBF, the rhodophyte cell is not membrane bound but found within the host cytoplasm (Lee & Anderson, 1991), and functions as an organelle, potentially facilitating energy transfer between symbiont and host (Hallock, 1999). This relationship could explain the adaptation of rhodophyte‐bearing species to a wide range of environmental conditions, from shallow areas (<5 m) with high water energy (Hohenegger *et al.,* 1999) to deeper areas in clear, oceanic conditions (up to 40 m) (Fujita, 2004). They can also be found in oligophotic conditions in coastal waters (Renema, 2018), are common and dominant in foraminiferal assemblages in hypersaline environments (Hallock, 1999), and in seagrass meadows (Reich *et al.,* 2015). However, details of the interaction between rhodophyte‐bearing hosts and their symbionts in relation to light intensity and other environmental conditions remain elusive (Ziegler & Uthicke, 2011). There is a clear lack of information on both chlorophyte‐ and rhodophyte‐bearing hosts' relationship with their respective symbionts. Given that there is evidence showing that dinoflagellate‐bearing hosts evolved from chlorophyte‐ and rhodophyte‐bearing hosts (Holzmann *et al.,* 2001), studies on the nutritional and physiological host–symbiont interactions might provide clues to the development of specific symbiont functions that contributed to the diversification of symbiosis within the Order Milliolida.

## IV. IDENTIFICATION AND POSSIBLE ROLES OF A PROKARYOTIC COMMUNITY

Organisms with photo‐symbiotic relationships with micro‐algae, such as reef‐building corals, giant clams, and sponges, also have a diverse array of bacterial associates with a role in maintaining the health of the holobiont (Ainsworth *et al.,* 2010). In these groups it has been demonstrated that the presence of photosynthetic symbionts influences the bacterial species composition, but not the species richness, evenness, or phylogenetic diversity of invertebrate‐associated microbiomes (Bourne *et al.,* 2013). The presence of both ecto‐ and endobiont bacteria that function as symbionts has been identified in sponges (Taylor *et al.,* 2007), reef‐building corals (Lesser *et al.,* 2004), and sea urchins (Guerinot & Patriquin, 1981).

Analogous to these better‐studied organisms, it is conceivable that prokaryotic endosymbionts perform a number of roles for prokaryotic endobionts in LBF, such as providing resilience to environmental variability, diseases, and in nitrogen fixation. Symbiosis with prokaryotes, especially nitrogen‐fixing bacteria, could significantly influence the ecology of their host (Fig. 1) and could have significant impacts on local nutrient biogeochemistry (Fiore *et al.,* 2010). Prokaryotes are ubiquitous in marine environments (Azam *et al.,* 1983; DeLong, 1992), and many species of benthic Foraminifera consume bacteria (Eubacteria, Archaea) as part of their diet (Bernhard & Bowser, 1992). Prokaryotic–foraminiferal associations are not uncommon. While some benthic foraminifera have associations with ectobionts, most foraminiferal–prokaryotic associations identified to date involve endobiont microbes (Bernhard, Tsuchiya, & Nomaki, 2018).

In Foraminifera, the presence of prokaryotic endobionts has been identified in very few non‐photosymbiotic species, but they could provide potential benefits such as supplying photosynthetically fixed carbon (Bird *et al.,* 2017), and aiding intracellular denitrification processes (Bernhard *et al.,* 2012). Only one study has identified intracellular red cyanobacteria through scanning electron microscopy, suggesting that it may be a potential endosymbiont of *Marginopora vertebralis* (Lee *et al.,* 1997). Similarly, Prazeres *et al*. (2017a) used next‐generation sequencing (NGS) to detect a high relative abundance of cyanobacteria associated with *Amphistegina lobifera* collected from oligotrophic environments, noting that the higher light availability in these environments could give the cyanobacteria a competitive advantage. As in corals, cyanobacteria could play a role in N‐fixation within the host when conditions are optimal (Lesser *et al.,* 2004). Other groups such as α*‐*Proteobacteria have been consistently identified in several LBF species through NGS (Bourne *et al.,* 2013; Webster *et al.,* 2016; Prazeres *et al.,* 2017a), and are commonly found as endobionts in reefs with high coral cover (Kelly *et al.,* 2014). The relative abundance of this bacterial class tends to decline significantly when populations are exposed to increases in sea‐surface temperature, when they are substituted by other bacterial taxa (Webster *et al.,* 2016). Natural populations of LBF exposed to environmental fluctuations show a similar pattern: the diversity of bacteria is higher than in physically and chemically stable habitats (Prazeres *et al.,* 2017a), and the relative abundance of Alpha‐Proteobacteria is generally low. Environmental variables such as water quality, temperature fluctuations and light exposure may help drive the observed compositional differences in the bacterial communities. Nonetheless, it is unknown whether the bacterial microbiome responds to, or is filtered by environmental gradients (Prazeres *et al.,* 2017a). Both environment and foraminiferal physiological state are likely to determine the intracellular prokaryotic community present in LBF (Bernhard *et al.,* 2018). The degree of dependence and the specific host–prokaryote relationships remain to be investigated. In LBF very little is known about the host–prokaryote–eukaryote relationship. Similar to transmission routes of algal symbionts, bacterial endobionts could be acquired either by vertical or horizontal transmission (Fig. 4). It is also likely that gametes could carry bacterial endobionts during sexual reproduction, as observed for sponges (Enticknap *et al.,* 2006).

## V. FOSSIL RECORD AND EVOLUTION OF MODERN LBF SPECIES

Algal endosymbiosis within Foraminifera has evolved multiple times. Even though we cannot determine past changes in the LBF microbiome, including both eukaryotic and prokaryotic associations because of the lack of preservation of the associates, we can use the morphology and spatial distribution of modern assemblages as an analogue (Renema, 2008b). Given the conservative presence of eukaryotic symbiont taxa in modern families, we can assume that similar symbionts were present in modern and extinct representatives of LBF families and their relatives.

### (1) LBF are effective trackers of climate change

LBF were important carbonate producers during warm periods over the past 400 My (Hallock & Glenn, 1986; Wilson & Rosen, 1998; Renema *et al.,* 2008; Morsilli *et al.,* 2012). Here we focus on the past 66 My, since this time interval includes the evolution of modern faunas. Following the Cretaceous–Paleogene (K–P) event, LBF began to stage a recovery in the Early Paleocene [∼66 million years ago (Ma)], resulting in increased size and the evolution of most of the Cenozoic (modern) LBF families. In this time interval, atmospheric CO_2_ concentrations were at least twice present levels, and sea‐surface carbonate saturation was significantly lower (Sloan & Rea, 1996; Zhang *et al.,* 2013). By the Early Eocene, six of the seven modern LBF families were already present (Serra‐Kiel *et al.,* 1998). The Calcarinidae are the only family that evolved during the (Late) Neogene (∼5 Ma; Renema, 2010).

The Cenozoic is characterised by a global cooling trend, interrupted by three warm intervals, the Paleocene–Eocene thermal maximum (PETM–EECO), the Middle Eocene climatic optimum (MECO), and the Middle Miocene climatic optimum (MMCO) (Zachos, Dickens, & Zeebe, 2008). During each of these warm intervals, rapid expansions of geographic ranges of LBF to higher latitudes are found in the fossil record. During the PETM–EECO, the diatom‐bearing species *Nummulites* occurred as far north as the Rockall bank at 57°N. Range expansions from the Paris Basin into the Belgium Basin are also associated with warm periods (King, Gale, & Barry, 2016; Baccaert, 2017). During the MECO, orthophragminids and *Nummulites* occured as far north as southern Alaska (55°N) and Belgium (51°N) (Adams, Lee, & Rosen, 1990). Following the Eocene–Oligocene cooling (Lear *et al.,* 2008), the latitudinal distribution of LBF contracted (Renema, 2008b). This trend was reversed during the MMCO, when geographic ranges expanded again, especially in the southern hemisphere. LBF became abundant as far south as southern Australia, which was positioned further south than at present. These excursions into higher latitudes were evolutionarily important for LBF. For example, a new species in the genus *Cycloclypeus* emerged during a range expansion, and replaced its ancestor following the subsequent range contraction during the Late Miocene (Renema, 2015).

### (2) The presence of algal symbionts matters

**Figure 6:**
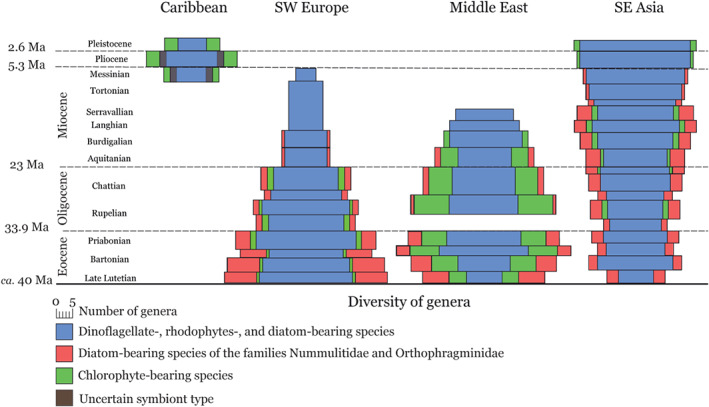
Spindle plots showing the diversity of symbiont‐bearing taxa throughout the Cenozoic [*ca*. 40–2.6 million years ago (Ma)]. Some fossil large benthic foraminifera (LBF) genera found in the Caribbean have uncertain symbiont type.

Temporal longitudinal trends in LBF diversity can be detected in tandem with global climatic patterns (Fig. 6). The most distinct is the difference in faunal composition between the West Atlantic and Tethyan realms. In the Tethyan realm, LBF diversity tracks the closure of the Tethys Ocean from west to east (Renema *et al.,* 2008). Distinct hotspots can be recognised: (*i*) in south‐west Europe during the Paleocene–Middle Eocene, (*ii*) in the Middle East from the Late Eocene to Early Miocene, and (*iii*) the present‐day Indo West‐Pacific biodiversity hotspot (Renema *et al.,* 2008). The taxonomic groups and symbiont types in which diversity is highest differ among these three hotspots (Fig. 6). In the south‐west Europe biodiversity hotspot, diatom‐bearing Nummulitidae and Orthophragminidae were especially diverse, including at least two families with numerous species. This is comparable to the present‐day Indo‐Pacific fauna, where Nummulitidae, Calcarinidae, and Amphisteginidae drive biodiversity patterns, and chlorophyte‐bearing species are rare Förderer, Rödder, & Langer (2018). Diversification in these provinces is primarily driven by adaptation to the depth (and light) gradient, and secondarily by onshore–offshore gradients (Hohenegger *et al.,* 1999; Renema, 2018). The Late Eocene–Early Miocene Middle Eastern biodiversity hotspot is dominated by genera housing chlorophytic symbionts and is thus more similar to the present day West Atlantic fauna, where LBF diversity is concentrated in the shallow photic zone. Based on modern analogues housing chlorophytic and dinoflagellate symbionts, which are found in relatively shallow environments (Waters & Hallock, 2017), it is likely that horizontal rather than vertical differentiation occurred in the Atlantic Ocean.

Dinoflagellate‐bearing taxa followed these trends to a much lesser extent. The Alveolinidae and Soritidae in the Eocene are comparable in their diversity and size distribution to the diatom‐bearing Nummulitidae in the Tethyan realm. During the Neogene the Soritidae diversified in the Caribbean (Hottinger, 2001) and Indo‐Pacific (Renema *et al.,* 2015). However, unresolved taxonomy impedes further inferences about the drivers of diversity in this group. In conclusion, the fossil record provides ample evidence that photosymbiosis has been a critical factor driving morphological diversity in LBF assemblages. Additionally, clear differences in spatio‐temporal distribution occur among taxa with different symbiont types, indicating that these are important for their adaptive potential with regard to environmental changes.

## VI. Importance of the microbiome to lbf ecology and distribution

The presence of eukaryotic and prokaryotic associates has fundamental implications for the adaptation and evolution of their host organisms and their responses to environmental change (Cavanaugh, 1994; Bourne *et al.,* 2016). Large‐scale geographic distribution patterns reveal that algal symbionts determine the distribution limits of LBF, such as rhodophytes in Australia, diatoms in southern Japan, and rhodophytes and dinoflagellates in the Mediterranean, indicating that there is no simple correlation with symbiont types (Fig. 3). At regional scales, the distribution of LBF is often restricted by large riverine outflows, which form physical and chemical barriers to dispersion (Langer & Hottinger, 2000). Furthermore, in turbid regions and in the eastern part of the Atlantic and Pacific Oceans, dinoflagellate‐ and chlorophyte‐bearing species are rare and diversity is low, indicating that diatom‐ and rhodophyte‐bearing taxa are more tolerant to higher nutrient levels (Hallock & Peebles, 1993; Renema, 2018). Additionally, light tolerance of algal symbionts strongly influences host depth distribution (Fig. 3). Taken together, this highlights the plasticity and capacity for adaptation of the host–algal symbiont system, as well as the need for a better understanding of how the holobiont functions, and the underlying mechanisms regulating bacterial and algal associations.

### (1) Stability and variability of microbial associates and LBF species occurrence

#### (a) *Persistent microbiome throughout the host's distribution range*

The biogeography and stability of the eukaryotic symbiont community are linked to the distribution of the host species (Fay, 2010), and species specificity is high (Garcia‐Cuetos *et al.,* 2005). For example, the diatom symbiont community of *Amphistegina gibbosa* (an Atlantic species) is continuous across its geographic range, but significantly different from the symbionts of the two Pacific species in the same genus, *A. lessonii* and *A. lobifera*, from Oahu, Hawaii, which also differ from each other (Barnes, 2016). Molecular studies showed that Pacific *A. lobifera* specimens that occur on the Great Barrier Reef (GBR) host eukaryotic symbiont communities similar to the Hawaiian population (Barnes, 2016; Prazeres *et al.,* 2017a). A recent molecular study also found no systematic differences in symbiont composition of *A. lobifera* populations between the Mediterranean and Red Seas (Schmidt *et al.,* 2016). It is plausible that in some LBF species, especially those with a circumtropical distribution such as *Amphistegina* spp., a stable, dominant eukaryotic symbiont over their entire geographic range would guarantee host functionality, regardless of their environment.

In the case of prokaryotic associations, organisms such reef‐building corals and sponges with a wide geographic distribution can form persistent associations with rare bacterial taxa, which can be species specific and are ubiquitous throughout the host's distribution range (Reveillaud *et al.,* 2014; Ainsworth *et al.,* 2015). This finding suggests the existence of strong ecological and/or evolutionary factors driving these associations (Reveillaud *et al.,* 2014), and a key role for bacteria in facilitating the success of host–algal symbioses across diverse environmental regimes (Ainsworth *et al.,* 2015). This hypothesis has never been tested in LBF, and it is unknown how conserved bacterial associations are. Nonetheless, it is plausible that a preserved core microbiome throughout the distribution of foraminiferal hosts is present. This pattern possibly explains why some species with conserved algal symbionts show a remarkable capacity to colonise a broad range of environments and can be found across a wide depth range.

#### (b) *Variable and diverse microbiome throughout their distribution*

In contrast to globally distributed LBF hosts, species with a more restricted distribution tend to show a more diverse and variable eukaryotic community across their distribution range (Pochon *et al.,* 2007). In this case, abiotic factors, rather than host identity, are predicted to have a higher influence on the symbiotic community, especially at local scales. Biogeographical barriers would define the availability of microbial associates (eukaryotic and prokaryotic), resulting in differences in the microbiome between the core and edges of their distribution. This pattern could be present along latitudes and with depth (Bongaerts *et al.,* 2015).

Patterns of microbiome variability are best observed in species that host dinoflagellates. Dinoflagellate‐bearing hosts exhibit high diversity in the Indian Ocean and West Pacific region, where symbionts are also more diverse than in the central Pacific, Red Sea (Gulf of Eilat), and Caribbean/Atlantic regions (Garcia‐Cuetos *et al.,* 2005). Symbiont community diversity can also be high in LBF species that live in marginal habitats characterised by high seasonal fluctuations in environmental parameters. In these locations mixed infections are common, and a high degree of flexibility can be found (Momigliano & Uthicke, 2013). A heterogeneous mix of symbionts would allow a host to select symbiont species from an existing pool that are better suited to environmental conditions at the extremes of the host's physiological limits (Fay, Weber, & Lipps, 2009).

Similarly, species with a narrow depth distribution, mainly restrained to shallow areas, exhibit higher flexibility and diversity of algal symbionts compared to species with distributions extending to deeper areas in both the Caribbean and Pacific (Baker, 2003). This is proposed to be a consequence of environmental heterogeneity in shallow environments, which are thought to be more variable both in space and time than deeper environments (Baker & Rowan, 1997; Baker, 2003). Additionally, in the case of LBF, shallow‐dwelling species select for symbionts that are more efficient at harnessing light, and as depth increases, specimens are likely to become less dependent on light for energy production, with a corresponding increase in reliance on heterotrophic feeding (e.g. Walker *et al.,* 2011).

Along with the diverse eukaryotic community, the bacterial microbiome may also vary across the hosts distribution range. The presence of a variable bacterial microbiome has been suggested to be advantageous when conditions change (Ziegler *et al.,* 2017). Bacteria can be utilised by the host to stabilise local host–algal symbioses, and the correlation between algal and bacterial associations can be strong. For example, dinoflagellate‐ and diatom‐bearing species show significantly different bacterial community compositions, even when specimens are collected from the same reef site and habitat (Bourne *et al.,* 2013). In this case, the identity of algal symbionts would drive the composition of the bacterial community (Webster *et al.,* 2016). In general, flexible, diverse recombination among hosts and associates is likely to be evolutionarily favoured over permanent associations. Host flexibility protects against extinction in a single host species (Langer & Lipps, 1995), particularly in the core of their vertical distribution (i.e. across depth) but also horizontally (i.e. with latitude and longitude) for LBF hosts with limited regional distributions.

#### (c) *Conserved algal community but variable bacterial associations*

It is predicted that conserved algal symbiont communities are particularly advantageous to LBF species living at the edge of species' geographical distribution, whereas variability in environmental conditions is accommodated by variable bacterial associations. For example, LBF and reef‐building corals living in tropical–subtropical transition zones exhibit low diversity, and a dominant symbiont type (Momigliano & Uthicke, 2013; Ng & Ang, 2016). Similarly, it has been shown that LBF hosts can form flexible and site‐specific associations with bacteria, while maintaining a conserved algal community, among populations exposed to different environmental conditions (Prazeres *et al.,* 2017a). The association of LBF with a range of different bacterial taxa could contribute to host distribution and survival across different habitats, as observed for reef‐building corals (Hernandez‐Agreda *et al.,* 2016). In this case, a variable bacterial community would assist LBF hosts to acclimate to specific environmental conditions, particularly when subject to high physical and chemical fluctuations (Prazeres *et al.,* 2017a).

These findings suggest that environmental filtering will differentially affect algal and bacterial symbiont communities: (*i*) by maintaining consistent, beneficial algal symbionts, and (*ii*) by acquiring local bacterial taxa, stabilising host–symbiont associations, and providing further capacity for local acclimation/adaptation (Shade & Handelsman, 2012). For example, the population of *A. lobifera* from the Red Sea has a higher thermal tolerance than an invasive population that has recently colonised the Mediterranean Sea (Schmidt *et al.,* 2016). However, both are capable of maintaining photosynthesis at 32 °C, which is well above the thermal optimum for most LBF species (Doo *et al.,* 2014; Prazeres *et al.,* 2017b), and support similar algal symbiont communities (Schmidt *et al.,* 2016). The presence of a local, diverse bacterial community that is responsive to biotic and abiotic processes could be responsible for the difference in thermal tolerance observed in the two *A. lobifera* populations. In this case, the bacterial microbiome acquired by the host assists with local acclimation of the migrant population during its colonisation of the Mediterranean Sea.

#### (d) *Flexible algal symbiont communities but conserved bacterial associations*

Mixed infection of symbionts has been reported in a phylogenetically broad diversity of hosts, especially those with constrained distributions (Fay *et al.,* 2009; Fay & Weber, 2012), as mentioned above. However, little is known about the relationship between highly variable algal symbiont communities and the bacterial microbiome. A variable bacterial community is suggested to be more advantageous in unstable environments (Prazeres *et al.,* 2017a). Therefore, it is plausible that in LBF hosts such as dinoflagellate‐bearing species, which rely to a great extent on their algal symbionts to meet their metabolic requirements (Fig. 1), flexibility in algal consortia is beneficial. In reef‐building corals, host identity appears to play a significant role in shaping bacterial communities (Brener‐Raffalli *et al.,* 2018). Similarly, LBF would also rely on a flexible algal community for resistance to thermal stress, and other environmental changes. As a result, an advantageous conserved bacterial community could be transferred across generations, while algal symbionts are acquired from the environment or actively selected by the host from its internal pool (Fay, 2010). This pattern could be common in dinoflagellate‐bearing species, as the pool of available symbionts in reef environments is high, given that other common reef organisms such as reef‐building corals and giant clams are also hosts of a diverse *Symbiodinium* community (Coffroth & Santos, 2005), which could potentially be acquired by LBF hosts.

### (2) The microbiome and the presence of cryptic speciation

In addition to selective pressures acting through bacterial and algal associates, the genetic diversity of the host can also provide mechanisms for responding to changes in environmental conditions. Genetic diversity within populations of LBF and their symbionts is poorly known, making it impossible to assess how genetic lineages within species (i.e. cryptic diversity) is distributed in space, and to test whether it is associated with different microbial associates. Speciation is not always accompanied by morphological change (Bickford *et al.,* 2007), and the presence of cryptic speciation in organisms that have been traditionally described based on morphological traits could hide genetic diversity within and among populations. Therefore, intraspecific genetic diversity is potentially a factor that could explain some host–symbiont specificity/variability within their distributional range.

Schmidt *et al*. (2016) identified genetic divergence of host *A. lobifera* populations between the GBR and Mediterranean/Red Seas, which was accompanied by different algal symbiont communities. It is plausible that, at the edge of their distribution, where biogeographic breaks occur, sexual reproduction occurs more frequently (Triantaphyllou *et al.,* 2012). This would allow individual hosts not only to acquire new symbionts through horizontal transmission, but would also lead to increased genetic diversity within LBF populations. Based on these observations, horizontal transmission is predicted to be advantageous during successful spatial expansion and to accommodate new environmental conditions, whereas at the core of their distribution vertical transmission of eukaryotic symbionts *via* asexual reproduction would be more prevalent (Rottger, 1974). Intraspecific genetic diversity may not only hide cryptic speciation but also host–symbiont specialisation (both eukaryotic and prokaryotic) across distributional ranges. In the latter, eukaryotic and prokaryotic associations could be adapted to specific climatic regimes, which could facilitate adaptation and geographic range shifts in response to climate change (Berkelmans & van Oppen, 2006). Potentially, each morphologically defined species could disguise a mosaic of biological (cryptic) species with divergent adaptive potential and microbial associations appropriate to the environment in which they live. Fitness trade‐offs in different environments could result in diversifying selection among populations invading different habitats, leading to divergence in temperature tolerance or life‐history adaptations, which could be driven by their microbiome (Shropshire & Bordenstein, 2016). Therefore, characterising the microbial communities associated with LBF will be a crucial step towards understanding how an invasive population can establish successfully as a dominant carbonate producer either: (*i*) by depending on the presence of a pre‐adaptive microbiome; or (*ii*) by re‐shaping the symbiont community. Given the relationship between LBF and their microbial associates, and the role that eukaryotic symbionts are known to play in LBF evolution (Lee & Hallock, 1987), it is likely that the microbiome, including prokaryotes and eukaryotes, could drive cryptic speciation in LBF.

## VII. FUTURE DIRECTIONS

We argue that the current predicted poleward expansion of some LBF species as global warming progresses partly hinges on their microbiome. Range expansions of LBF have triggered substantial changes in ecosystem function, including shifts in species diversity, carbonate production, and ecological impacts on native biota (Langer & Hottinger, 2000; Langer, 2008; Weinmann *et al.,* 2013a,b *;* Langer *et al.,* 2013b). The potential for the recombination of different eukaryotic and prokaryotic partners (Fig. 4), and natural selection for host populations associated with more tolerant symbionts may serve to create communities of holobionts suited to altered environmental conditions. Consequently, symbiont communities might assist LBF species to respond to ongoing climate change.

The geological record demonstrates that LBF can be used to trace expansions of subtropical and tropical belts during climate warming, with regional differences in the dominant eukaryotic symbiont types. Northern and southernmost records during warm intervals relate to fossil and modern diatom‐bearing taxa, especially nummulitids, orthophragminids, and lepidocyclinids (Figs 3 and 6). The diversity and abundance of LBF has varied with space and time, while modern assemblages show similar patterns of biogeography and geographical range expansion driven by current trends of ocean warming, as seen throughout the Cenozoic (Fig. 3). For example, in the Caribbean region, *Amphistegina* or *Archaias* are typically the dominant LBF taxa (e.g. Baker *et al.,* 2009), and the diversity of chlorophyte‐bearing species is high. By contrast, on the GBR and in the Indo‐Pacific, LBF diversity is higher among the diatom‐bearing hyaline taxa, which tend to be the dominant species in oligotrophic reef‐associated environments. Diatom‐bearing species have dominated shallow platforms throughout the Cenozoic (Wilson *et al*., 1998; Morsilli *et al.,* 2012; Novak & Renema, 2018), and are good candidates to become the dominant calcifiers in carbonate environments in the future (Weinmann *et al.,* 2013a). This is largely due to their high tolerance to a broad range of temperature, nutrient, and light levels (Weinmann *et al.,* 2013a *;* Langer *et al.,* 2013b *;* Prazeres, Uthicke, & Pandolfi, 2016b *;* Prazeres *et al.,* 2017b), as well as their proven capacity to colonise new habitats and areas efficiently. We argue that the combination of a comparatively stable relationship with eukaryotic symbionts, and a highly flexible relationship with prokaryotic endobionts underpins this capacity. Nonetheless, despite the importance of prokaryotes for survival and adaptation in other organisms (Apprill, 2017), there remains very little information about bacterial communities associated with LBF. Future studies on the biology, ecology, and evolution of LBF should take into consideration the role of prokaryotic associates in facilitating and/or mediating species responses to changes in environmental conditions and colonising new environments. In light of this, some specific lines of research should be considered: (*i*) re‐assessment of eukaryotic symbiont diversity using molecular techniques in addition to morphology, specifically in the context of environmental gradients. Even though the identities of major algal groups hosted by LBF families are relatively well known, there are few studies on the intra‐ and interspecific distribution of eukaryotic symbionts, biogeographical breaks, and biotic and abiotic factors that influence the flexibility/specificity and diversity of these associations. (*ii*) Assessment of the diversity of bacterial communities associated with LBF, utilising next‐generation sequencing, across gradients of depth, latitude, and longitude. Quantifying functional shifts in bacterial communities, and how bacteria contribute to the energy budget and other physiological pathways will also represent important advances in LBF biology. The scarcity of data on the role of prokaryotes in LBF is a significant knowledge gap, and should be a high priority for future research. (*iii*) Identification of bacteria using imaging techniques such as fluorescent *in situ* hybridisation and electron scanning microscopy, which should help decipher the potential roles of bacteria in LBF intracellular space. (*iv*) Further research on neglected eukaryotic symbiont groups such as rhodophytes, chlorophytes, and chrysophytes. Rhodophyte‐bearing LBF are a particularly tantalising model, as not only do they show a peculiar relationship with their symbionts, but most species that host red algae are cosmopolitan and occupies a wide range of environments. (*v*) Study the presence of cryptic diversity and phylogeography of species that have been previously described based on morphological characteristics. Genetic differences are not necessarily accompanied by the development of morphologically divergent traits (Darling & Wade, 2008), and the presence of cryptic diversity may shed light on biogeographic boundaries of eukaryotic and prokaryotic symbiont communities, as well as the abiotic factors that drive host–symbiont specialisation.

## VIII. CONCLUSIONS

(1) Geological and modern records of LBF distributions show that diatom‐bearing taxa are the most common, abundant, and dominant taxa across wide environmental gradients, whereas dinoflagellate and chlorophytic species tend to be more restricted in their distribution, and less tolerant to nutrients and terrestrial influences.

(2) Modern, cosmopolitan diatom‐bearing species depend less on their eukaryotic microbes for meeting their energetic requirements and show stable diatom symbiont communities in the core of their distribution. By contrast, dinoflagellate‐bearing taxa, are more reliant on their symbiont for survival, and tend to have a flexible association responsive to environmental conditions across their range of occurrence.

(3) The occurrence of cryptic speciation in many LBF species can hide host–symbiont specialisation and adaptation capacity of the host to different environments. The capacity of species to adapt to their new environment is a critical component for understanding the role of evolutionary processes in the assembly and dynamics of natural communities.

(4) Abiotic factors (e.g. temperature, water clarity, and nutrient availability), and eukaryotic symbionts had an important synergistic contribution to the expansion and contraction of LBF distribution during warming–cooling cycles during the Cenozoic.

(5) Interactions between the host, the eukaryotic symbionts, and the prokaryotic endobionts is key to understanding the plasticity, adaptive potential, and resilience of LBF to environmental change. Additionally, the physiological state of both the host and the associates is likely to influence the identity and diversity of the eukaryotic and prokaryotic community.

(6) In recent years, we have seen major advances in describing and understanding the role of microbial assemblages in reef fauna, including reef‐building corals, sponges, and benthic Foraminifera, and the role that bacteria and other microorganisms play in maintaining the health of the reefs. LBF are essential ecosystem engineers and prolific carbonate producers, and the study of their microbiome should provide important information on their ability to respond to climate change.

(7) Identifying host–prokaryote–eukaryote associations and genetic structure within LBF host populations is crucial to a better understanding of the capacities of LBF species to adapt to their new environment or to shift their distribution range. These are critical components for understanding the role of evolutionary processes in shaping the assembly and dynamics of natural communities.
